# Correlation of sPD1 with Procalcitonin and C-Reactive Protein Levels in Patients with Sepsis

**DOI:** 10.22074/cellj.2021.6941

**Published:** 2021-03-01

**Authors:** Zahra Bakhshiani, Saloomeh Fouladi, Samaneh Mohammadzadeh, Nahid Eskandari

**Affiliations:** 1 Department of Immunology, Faculty of Medicine, Isfahan University of Medical Sciences, Isfahan, Iran; 2 Poursina Hakim Digestive Diseases Research Center, Isfahan University of Medical Scineces, Isfahan, Iran; 3 Applied Physiology Research Center, Isfahan University of Medical Sciences, Isfahan, Iran

**Keywords:** C-Reactive Protein, Procalcitonin, Sepsis, sPD1

## Abstract

**Objective:**

Sepsis results from dysregulated host responses to infection, and it is a major cause of mortality in the world. Co-inhibitory molecules, such as PD-1, play a critical role in this process. Considering the lack of information on the relation between sPD1 and sepsis, the present study aimed to examine the sPD1 level in septic patients and evaluate its correlation with procalcitonin (PCT) and C-reactive protein (CRP) levels.

**Materials and Methods:**

This descriptive cross-sectional study consisted of three groups, including septic patients (n=15), suspected of sepsis (n=15), and healthy subjects (n=15). White blood cells (WBCs) and platelet (PLT) counts are evaluated. The serum levels of CRP, PCT, and sPD1 were measured by immunoturbidimetric assay, electro- chemiluminescence technology, and the enzyme-linked immunosorbent assay (ELISA), respectively.

**Results:**

Our study indicated that there was a significant difference in WBC and PLT counts between the septic group compared to suspected and control groups (P<0.001, P<0.01, respectively). The CRP level was significantly higher in septic compared to suspected and control groups (P<0.001). There was also a significant difference between the PCT level in septic and suspected groups in comparison with the controls (P<0.001, P<0.01). The sPD1 level was significantly higher in septic patients compared to suspected and control groups (P<0.001). In septic patients, sPD1 levels were correlated positively with the CRP and PCT levels.

**Conclusion:**

Overall, sPD1 correlation with inflammatory markers, might propose it as a potential biomarker to sepsis diagnosis. However, the clinical application of serum sPD-1 testing in patients with sepsis requires further investigation.

## Introduction

Sepsis refers to a life-threatening dysfunction of the organ that is caused by a dysregulated response of the host to the infection and, if not controlled, may become the severe form, septic shock. Sepsis is one of the most important causes of morbidity and mortality all over the world and often requires urgent and supportive treatment in the intensive care unit (ICU) due to the involvement of several organs. About 18 million new cases of sepsis are reported each year, with a mortality rate of 30-50% (1).

Clinical symptoms of sepsis include tachycardia, tachypnea, fever, leukocytosis, etc. Severe Sepsis is associated with hypoperfusion, organ dysfunction, or hypotension (2).

In sepsis, invasion of the microorganisms to the bloodstream occurs, so that they localize and proliferate and release their pathogenic factors into the bloodstream. These products can stimulate the release of endogenous sepsis mediators from endothelial cells, neutrophils, monocytes, macrophages, and plasma cell precursors (3, 4).

Traditionally, sepsis was considered as an excessive systemic proinflammatory reaction to invasive microbial pathogens. More recently, it has been suggested that the early phase of hyperinflammation is followed or overlapped by long-term immunosuppression, considered as sepsis-induced immunoparalysis. The immunoparalytic status is determined by impaired innate and adaptive immune responses and may play an important role in the pathogenesis of multiple organ failure, tissue damage, and death caused by sepsis (5, 6).

Early diagnosis and immediate anti-microbial therapy in the treatment of sepsis is essential in order to save the patient’s life. In addition to clinical evaluations, laboratory hematologic, microbiological, and immunological tests are needed to diagnose sepsis (7, 8).

Many cytokines or other proteins have been studied as potential biomarkers to determine a hyperinflammatory status in patients with sepsis. From these, C-reactive protein (CRP) and procalcitonin (PCT), white blood cells (WBC), interleukin-1 (IL-1) and IL-6 can be referred. Compared with CRP, the PCT test has a higher diagnostic and prognostic value and can differentiate bacterial and viral meningitis (9, 10).

Immune system suppression is one of the major causes of mortality in patients with severe sepsis (11, 12). Negative co-stimulatory molecules play an important role in the immune system function and regulate cell proliferation, differentiation, and apoptosis negatively. 

With knowledge of the mechanism of the immune response in sepsis, several immunosuppression markers are proposed, such as the superfamily B7- CD28 called programmed cell death 1 (PD-1) so that PD1 and programmed death ligand-1 (PD-L1) inhibit the function of B and T cells (13, 14). 

PD1 has two forms, a form bind to the membrane, and another soluble form, called soluble PD-1 (sPD-1). sPD-1 is encoded by PD-1Dex3. It has no transmembrane region, and it has a biological function. sPD-1 could enter the bloodstream so that it can perform its function in the immune response (15). 

Considering the limited information available concerning the relation between sPD-1 and sepsis, this study aimed to investigate the serum sPD-1 value as an immunosuppressive phase marker compared to CRP and PCT levels in recognized sepsis, suspected sepsis, and healthy subjects. Moreover, the relation between the sPD1 level and these two inflammatory markers was studied.

## Materials and Methods

### Study subjects

In a descriptive cross-sectional study, patients (n=30) who admitted to medical or surgical ICUs FatemaZahra hospital in Najafabad-Isfahan (from October to December 2019), who were older than 18 year of age, and fulfilled a consensus panel definition of sepsis were included in the study. Sepsis was defined as the presence of systemic inflammatory response syndrome and a known or suspected source of infection. The exclusion criteria were including bone marrow irradiation, chemotherapy, or radiation therapy within the past six months, human immunodeficiency virus (HIV) infection or viral hepatitis, and consumption of immunosuppressive medications. 

Before initiation of antibiotic therapy in suspected of sepsis patients, whole blood was taken from the subjects for blood culture (3-4 ml), complete blood count (CBC) (1-2 ml), PCT, CRP, and sPD1 measurements (2-3 ml). Serum was separated from blood cells by centrifugation and stored in 3 plastic tubes at -20˚C for measurements of PCT, CRP, and sPD1 levels. 

Finally, according to clinical symptoms of sepsis, microbiologic and laboratory results,

patients categorized into two groups: 1. Proven sepsis (n=15) with clinical symptoms of sepsis and positive blood culture test and 2. Suspected sepsis (n=5) with clinical symptoms but negative blood culture result.

Healthy volunteers (n=15) were recruited as healthy controls. All the control subjects were age- and sexmatched. The study protocol was confirmed by the Ethics Committee of Isfahan University of Medical Sciences (Code of Ethics: IR.MUI.REC.1384012). Written consent was obtained from all subjects or their families.

### Hematological examination

A CBC is performed on the automated hematology analyzer KX-21 (Japan) using the study participants’ ethylenediaminetetraacetic acid (EDTA, VACUTEST KIMA, Italy) blood tubes, which are obtained via the phlebotomy component. WBCs and PLT counts are the most important parameters in the sepsis.

### Microbiological examination

Four ml of blood was added to blood culture media (Biphasic) and incubated at 37˚C for 5-7 days. The positive blood cultures media were sub-cultured on blood agar (Himedia, India) and EMB media. The isolated microbes were identified by standard bacteriological methods.

### Measurements of C-reactive protein, Procalcitonin, and sPD-1 levels

For the quantitative determination of CRP in serum, latex particle enhanced immunoturbidimetric assay was performed using the Mindray BS- 400 analyzer (China). 

Latex particles coated with an antibody specific to human CRP clumps in the presence of CRP in the serum sample forming immune complexes. The intensity of the scattered light is proportional to the CRP level in the serum. The light scattering is measured by reading turbidity (absorbance) at 570 nm. The CRP concentration is determined via a calibration curve. 

The lower limit of detection was 0.1 mg/L, and the expected value for CRP in healthy individuals was below 6.2 mg/L.

The serum level of PCT was measured using the electro-chemiluminescence (ECL) technology (Roche Diagnostics, Germany). Related concentrations were measured according to protocols using an immunoassay analyzer. The lower detection limit was 0.02 ng/mL. 

Through the first incubation, antigen in the sample, a biotinylated monoclonal PCT‑specific antibody, and a monoclonal PCT-specific antibody labeled with a ruthenium complex react to form a sandwich complex. During the second incubation, by the addition of streptavidin-coated microparticles, the complex becomes bound to the solid phase by the interaction of biotin and streptavidin. Then the reaction mixture is aspirated into the measuring cell where the microparticles are magnetically captured onto the surface of the electrode. Unbound substances are then removed with ProCell/ProCell M. Then, the application of a voltage to the electrode induces chemiluminescent emission, which is measured by a photomultiplier.

Finally, for determining the results, a calibration curve which is specific to the instrument generated by 2‑point calibration and a master curve provided via the reagent barcode.

The concentration of sPD1 was measured by the ELISA according to the kit protocol (DuoSet Human PD-1, R&D systems, Minneapolis, MN, USA) on an automatic microplate reader (Stat Fax 2100, USA). The detection range of the kit was 156-10000 pg/ml.

In brief, high bind microtiter plates were incubated with the capture antibody, sealed, and incubated overnight. On the next day, plates were washed (3 x with phosphate buffered saline (PBS) containing 0.05% Tween). Then, 300 µL /well bovine serum albumin (BSA) (1% in PBS) was added as a blocking agent. The plates were incubated at room temperature for 1 h. After a washing step, calibrators or patient samples were added, sealed, and incubated at RT for 2 hours. For the preparation of the calibration curve, 1:2 dilutions of the standard ranging from 10 ng/mL to 156 pg/mL, was used. After the washing step, the detection antibody was added, sealed, and incubated for 2 hours. Once again, plates were washed, and Streptavidin-HRP was added and incubated for 20 minutes. After the last washing step, the substrate solution was added to each well and incubated for 20 minutes at RT. Then stop solution was added to each well. Finally, absorbance was read at 450 nm with wavelength correction set at 540 nm.

### Statistical analysis

Data analysis was conducted using IBM SPSS 21 statistics (IBM, USA). Values are represented as mean ± standard deviation (SD). Shapiro-Wilk Normality test was performed to confirm the normality of data distribution. The difference between the groups was examined through the one-way analysis of variance (ANOVA) test along with the Tukey HSD post hoc. The Chi-square test was used to compare the qualitative variables. Pearson’s correlation coefficient test was used to assess the strength of the correlation between the sPD1 and other variables. The level of statistical significance was set at P<0.05.

## Results

### Characteristics of the patients

In this study, Blood cultures were positive for all patients. The identified bacteria included Staphylococcus aureus (n=3) Streptococcus betahemolytic group A (n=3), Escherichia coli (n=2), Klebsiella pneumoniae (n=5), and Enterobacter (n=2). The age and sex distribution in the proven, suspected sepsis, and control groups are shown in Table 1.

### Hematological examination

This study evaluated WBC and platelet counts in different groups. Results showed that there was a significant difference in WBC counts between the septic and suspected groups compared to healthy controls (P<0.001). There is also a significant difference between septic and suspected groups (P<0.01).

There was a significant difference in PLT counts between septic group compared to suspected and control groups (P<0.01). The results are summarized in Table 2.

**Table 1:** Characteristics of the study groups


Parameter	Septic patients	Suspected group	Control group	P value

Number	15	15	15	
Age (Y)	48.46 ± 17.6	41.66 ± 19.38	44.4 ± 14.8	>0.05
Gender (M/F)	8/7	9/6	10/5	>0.05


P>0.05 compared to the controls. Data indicated as mean ± standard deviation (SD) (n=15 per group).

**Table 2:** Variable values of the study groups


Parameter	Septic patients	Suspected group	Control group

WBC (×10^3^/µl)	14.7 ± 6.53	9 ± 2.3	5.4 ± 0.86
PLT (×10^3^/µl)	178.66 ± 74.97	263.67 ± 65.171	267 ± 76.075
CRP (mg/L)	50.97 ± 11.4	29.54 ± 16.9	4.38 ± 2.04
PCT (ng/ml)	4.55 ± 2.2	2.2 ± 1.4	0.19 ± 0.1
sPD1 (pg/ml)	195.1 ± 151.7	23.9 ± 12.3	13.1 ± 7.8


Data indicated as mean ± standard deviation (SD, n=15 per group). WBC; White blood cells, PLT; Platelet, CRP; C-reactive protein and PTC; procalcitonin.

### Serum levels of C-reactive protein, Procalcitonin, and sPD1

This study evaluated the CRP, PCT, and sPD1 serum levels in different groups. There was a significant difference between the mean of CRP level in septic patients and the suspected group compared to healthy controls (P<0.001). In addition, it was observed a significant difference between septic and suspected groups (P<0.001).

Results showed that the PCT level was significantly higher in septic and suspected groups in comparison with the controls (P<0.001, P<0.01, respectively). There was also a significant difference between the mean of PCT level in septic compared to the suspected group (P<0.01).

**Fig.1:**
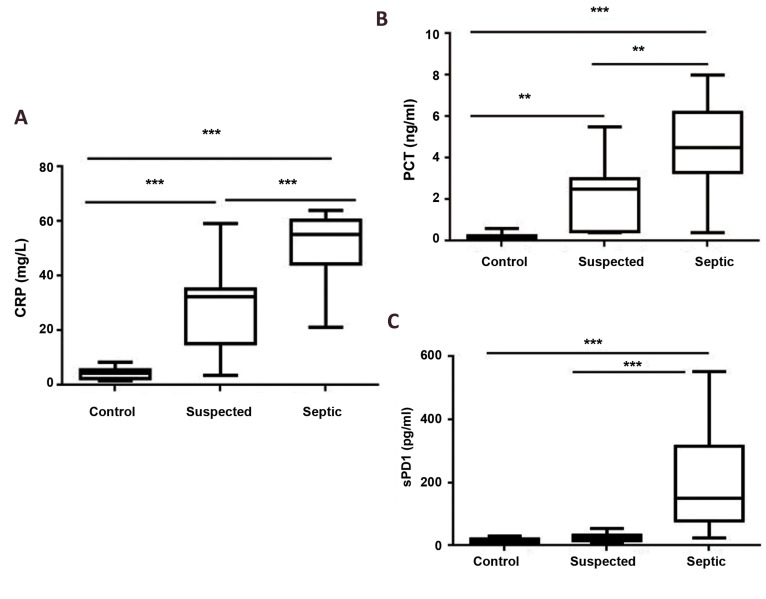
Comparison of serum levels of CRP, PCT, and sPD1 in the different groups. **A.** There was a significant difference between the mean of CRP level in septic patients and the suspected group compared to healthy controls. **B.** PCT level was significantly higher in septic and suspected groups in comparison with the controls. **C.** There was a significant difference between the sPD1levels in septic patients compared to suspected and control groups. Procalcitonin. Data indicated as mean ± standard deviation (SD, n=15). CRP; C- reactive protein, PCT; **; P<0.01, and ***; P<0.001.

The results showed that there was a significant difference between the sPD1 levels in septic patients compared to suspected and control groups (P<0.001). But there was not a significant difference between the mean of sPD1 level in suspected compared to the control group (P>0.05). The results are shown in Table 2 and Figure 1.

### Sensitivity, specificity, positive predictive value, and negative predictive values

The optimum cut-off value was found to be 6.2 mg/l for CRP, 0.5 ng/ml for PCT, and 42 pg/ml for sPD1. At cutoff values, sensitivity, specificity, PPV, and NPV values of these parameters were calculated for the diagnosis of sepsis, and the results are shown in Table 3. 

**Table 3:** Sensitivity, specificity, PPV, and NPV values of serum variables


Variable	Sensitivity (%)	Specificity (%)	PPV (%)	NPV (%)

CRP (mg/L)	80	86.6	85.7	81.2
PCT (ng/ml)	66.7	86.7	83.3	72.2
sPD1 (pg/ml)	60	93.3	90	70


PPV; Positive prediction value, NPV; Negative prediction value, CRP; C reactive protein, and PCT; Procalcitonin

### Correlation among serum sPD-1 and other parameters

**Fig.2:**
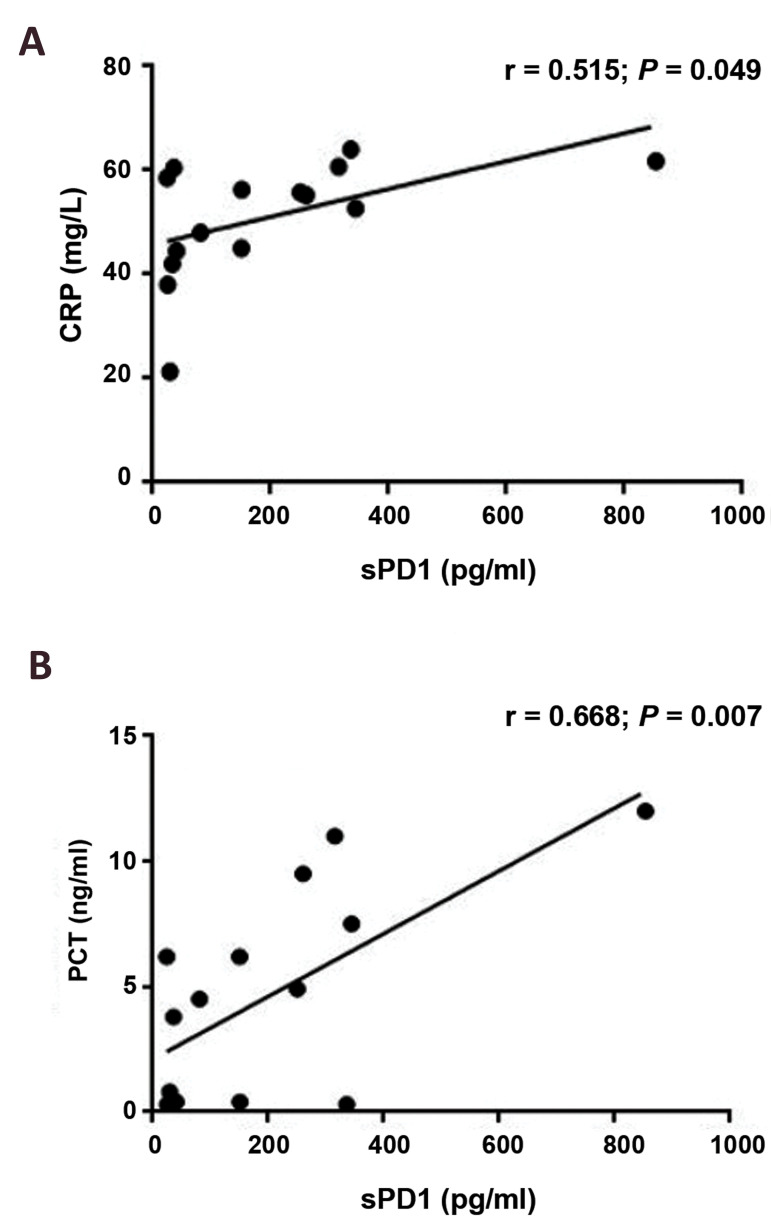
The correlations among sPD1, CRP, PD1, and PCT levels in septic patients. There was a significant positive correlation between the serum level of **A.** CRP and **B.** PCT with sPD1 in patients with sepsis (n=15). CRP; C-reactive protein and PCT; Procalcitonin.

The result showed that there was a significant positive correlation between the serum sPD1 level and values of serum PCT and CRP in the septic patients (r=0.668, P=0.007; r=0.515, P=0.049, respectively), Figure 2. However, no correlation detected between sPD-1 levels and age, WBC or PLT counts in all three groups (P>0.05).

## Discussion

In the present study, we found that sPD-1 levels in patients with sepsis were higher than those who were suspected of sepsis and healthy controls. Also, sPD1 levels were positively correlated with PCT and CRP levels. These findings suggest that this increased level might propose sPD-1 as a potential bio-marker to sepsis diagnosis.

Immune dysfunction is regularly attendant with an increased risk of death from sepsis. The latest studies indicated that pro- and anti-inflammatory reactions occurred in sepsis concurrently, even in the early stage. With knowledge of the mechanism of immunologic response at a different stage of sepsis, it seems immunosuppression is thought-out to be the main factor affecting the outcome of septic patients (16, 17)

PD‑1 is one of the best known co‑inhibitory molecules. It has been observed in studies that the expression of this molecule on macrophages and peripheral blood monocytes was increased in a mouse model of sepsis, and the administration of PD1 antagonist has improved the survival of the infected animal (18, 19). According to the results of a clinical study in recent years, PD-1 expression on the surface of T lymphocytes in patients with sepsis was significantly increased (20). The inhibition of the PD-1/PD-L1 pathway with anti-PD-1 and antiPD-L1 antibodies reduces sepsis-induced apoptosis in lymphocytes and returns the ability of immune cells to produce proinflammatory cytokines (20, 21).

The overexpression of PD-1 on the T lymphocytes or changed sPD-1 levels has been observed in patients with aplastic anemia (22), immune thrombocytopenia (ITP) (23), rheumatoid arthritis (RA) (24), or cancer (25, 26).

There are only a few studies demonstrating sPD1 levels in sepsis.

Zhao et al. (27) described that sPD-1 levels were increased in sepsis patients, and its value has also increased with increased severity of the disease. Their study showed that sPD-1 was an independent risk factor for the 28-day mortality of septic patients. Thus, sPD-1 may be used as an immunological biomarker for early assessment of the severity and prognosis of sepsis. 

Yongzhen Zhao et al. (28) in another study found that peripheral blood levels of sPD-1 and sPD-L1, PD-1 expression on CD4^+^ T cells and CD8^+^ T cells and PD-L1 expression on monocytes are higher in non-survivors than in survivors sepsis, and the levels of sPD-1 and sPD-L1 have a correlation with the severity of the disease. They conclude that monitoring the concentrations of sPD-1 may improve the prognostic assessment in septic patients during the first week of treatment. 

We also found that sPD-1 levels in patients with sepsis were higher than suspected and control groups. Despite the similarity of our result in this regard with other studies, the results of Lange et al. (29) study showed that sPD-1 values in patients with sepsis and septic shock were lower than control people and not related to the severity of the disease. This difference may be due to the level of sPD-1 in healthy control is higher than healthy subjects in our study, which should be further investigated in different populations. Another cause of this discrepancy may be related to differences in patient characteristics, such as age, sex, race, or even sampling time and disease status. 

However, the exact function of the sPD-1 is not well known in sepsis. The increase in membrane-bound PD-1 may lead to a secondary increase in sPD-1 levels. It seems that similar to rheumatoid arthritis patients, PD-1 and PD-L1 overexpressed, and the sPD-1/sPD-L1 concentrations also increased to prevent the regulatory effect of membrane-bound PD-1 and PDL1 (28).

It should be noted that the detection of serum sPD-1 is easier than the detection of membrane-bound PD-1 by flow cytometry and accelerates the diagnosis of disease in clinical applications.

CRP is a conventional marker used for diagnosis of sepsis and inflammation, In this study, as well as similar studies, the concentration of CRP was higher in septic and suspected compared to healthy controls (30, 31). Although some studies have shown that inflammatory factors such as CRP increase with age, in this study, the average age of participants is less than 50 years, and thus, the effect of age disappears (32).

In this study, like most similar studies, PCT levels also were significantly increased in septic and suspected patients compared to control subjects (33, 34). PCT concentrations slightly increased in bacterial infections without a systemic inflammatory response, like localized infections (35). Maybe that’s why in our study, PCT levels were significantly higher in septic compared to suspected of sepsis patients.

In the study conducted by Zhao et al. (27), they found that as the disease progressed, the levels of sPD-1, CRP, and PCT gradually increased. But in another study performed by them, CRP and PCT as the inflammatory markers showed no significant correlation with sPD-1/ sPD-L1 (28).

Contrary to this study, our results showed that the sPD1 level is positively correlated with the level of CRP and PCT in patients with sepsis. Differences in the severity of disease in the patient sample could also have contributed to the differences. Our study selected patients with general sepsis, while Zhao et al. (28) selected severe sepsis and septic shock patients. Different methods of assessment also should be considered. 

The sPD-1 had a higher PPV and NPV values in patients with sepsis, the specificity was higher, but the sensitivity was low. These findings are consistent with the results of a similar study and could indicate the role of sPD-1 in the diagnosis of sepsis (28). 

This study had a few limitations. We measured only the sPD-1 level, and the expression of PD-1 / PD-L1 and sPDL1 was not measured. Patient follow-up was not carried out, and the sPD-1 level changes were not evaluated during the disease. The severity of the disease was not graded accurately, and we could not evaluate the correlation of other variables with the severity of the disease. Finally, this study was conducted at a center only, and further studies are needed larger sample size to confirm the results of the study

## Conclusion

Overall, the serum levels of sPD-1 were significantly increased in patients with sepsis. The serum sPD-1 levels were positively correlated with the CRP and PCT levels in septic patients. This test was more specific than these two markers. The sPD1 correlation with inflammatory markers might propose it as a potential biomarker for the sepsis diagnosis. However, the clinical application of serum sPD-1 testing in patients with sepsis requires further investigation.
